# Outcomes of pediatric kidney re-transplantation: a single-center cohort study

**DOI:** 10.1038/s41598-025-28562-w

**Published:** 2025-11-27

**Authors:** Franziska Meyer, Hans-Michael Tautenhahn, Sophie Seiffer, Jan Kowald, Juliane Putz, Hans-Jonas Meyer, Svitlana Ziganshyna, Michael Pohl, Katalin Dittrich, Daniel Seehofer, Uwe Scheuermann

**Affiliations:** 1 Department of Visceral, Transplantation, Vascular and Thoracic Surgery, University of Leipzig Medical Center, Liebigstrasse 20, 04103 Leipzig, Germany; 2 Medical Department III, Division of Nephrology, University of Leipzig Medical Center, Leipzig, Germany; 3 Department of Urology, University Hospital Carl Gustav Carus, TU Dresden, Dresden, Germany; 4 Department of Diagnostic and Interventional Radiology, University of Leipzig Medical Center, Leipzig, Germany; 5 Organ Donation Coordinator Unit, University of Leipzig Medical Center, Leipzig, Germany; 6 KfH Kidney Center for Children and Adolescents, St. Georg Hospital, Leipzig, Germany; 7 Division of Pediatric Nephrology and Transplantation, Department of Pediatrics, University of Leipzig Medical Center, Leipzig, Germany

**Keywords:** Pediatric kidney transplantation, Re-transplantation, Outcome, Survival, Diseases, Medical research, Nephrology

## Abstract

Children with end-stage renal disease (ESRD) frequently require more than one kidney transplantation (KT) during their lifetime due to limited graft longevity. Despite this clinical reality, few studies have evaluated long-term outcomes following repeat pediatric KT. We conducted a retrospective single-center study analyzing 120 KTs performed in 89 pediatric recipients between 1993 and 2024. Outcomes included graft function, postoperative complications, and long-term graft and patient survival. Recipients were stratified into primary (1KT), second (2KT), and third (3KT) transplantation groups. At the time of 1KT, median recipient age was 11.0 years (IQR 7.0, 14.5). Living donation accounted for 16.7% of procedures. Graft failure within five years occurred in approximately 20% of 1KT cases. Half of these patients received a 2KT after a median waiting time of 4.6 years (IQR 2.1, 9.0). Rates of early postoperative complications and kidney function were comparable across groups. Kaplan–Meier analysis revealed significantly improved long-term survival following 2KT compared to failed 1KT (*p* = 0.023). Repeat kidney transplantation is a feasible and effective strategy for pediatric ESRD patients. Second transplants provide long-term outcomes comparable to, or better than, initial grafts. Multicenter prospective studies are warranted to confirm these findings.

## Introduction

End-stage renal disease (ESRD) in children is a rare condition, with an estimated incidence of approximately 9 cases per million age-related population. The leading causes include congenital anomalies of the kidneys and urinary tract (CAKUT) and primary glomerular diseases, such as focal segmental glomerulosclerosis (FSGS)^1–3^.

Children affected by ESRD often experience growth retardation, delayed neurocognitive development, impaired quality of life, and an increased risk of cardiovascular disease and premature mortality^1,3–5^. Kidney transplantation (KT) is considered the treatment of choice for pediatric ESRD, as it supports age-appropriate development, enhances quality of life, and significantly improves long-term survival compared to dialysis^6–9^.

Despite these benefits, the limited longevity of kidney allografts often necessitates multiple transplantations throughout a patient’s life. However, long-term follow-up data for pediatric KT remain limited, partly due to disruptions in care following transition to adult services. Although multicenter registries provide aggregate long-term data, they frequently lack detailed information on perioperative management and early graft function.

This study investigates clinical outcomes following pediatric KT—including repeat transplantation—at the University Hospital of Leipzig between 1993 and 2024. The aim is to provide granular insight into long-term graft and patient survival, perioperative complications, and the feasibility of repeated KT in a well-characterized pediatric cohort.

## Patients and methods

### Data collection and study population

This retrospective single-center study included all pediatric patients (< 18 years) who underwent kidney transplantation (KT) at the University Hospital of Leipzig between October 1993 and August 2024. In nine cases, the primary KT was performed at an external center. Follow-up data were collected until September 2025. The study was approved by the Ethics Committee of the University of Leipzig (approval number: 111/16/14032016) and conducted in accordance with the Declaration of Helsinki. According to §34 of the Saxon Hospital Act (Sächsisches Krankenhausgesetz), all patients provided a general broad consent upon hospital admission permitting the anonymized use of their data for research unless revoked. Given the retrospective design and anonymized data analysis, the Ethics Committee waived the need for additional written informed consent. No organs were procured from prisoners. All organs were procured while taking care to not violate the privacy of donors.

Patients received pre- and post-transplant care at the KfH Kidney Center and the Department of Pediatrics, St. Georg Hospital, Leipzig. Transplant evaluations were conducted at the University Hospital of Leipzig, and all transplant surgeries were performed by the Department of Visceral, Transplant, Thoracic, and Vascular Surgery. Postoperative care was provided by the University Children’s Hospital Leipzig, followed by long-term follow-up at the KfH Kidney Center. For patients transferred to other institutions, follow-up data were collected with written informed consent.

Collected data included recipient and donor demographics (age, sex, body mass index [BMI]), cause of ESRD, dialysis history, duration on the Eurotransplant waiting list, comorbidities, and transplant-related parameters such as human leukocyte antigen (HLA) mismatches, panel reactive antibody (PRA) levels, cold ischemia time (CIT), warm ischemia time (WIT), and operative time.

Surgical techniques were tailored to patient anatomy and body size. Children weighing less than 20 kg typically received grafts with aorto-caval anastomoses. Older recipients underwent iliac vessel anastomoses. To prevent postoperative abdominal compartment syndrome, intraoperative assessment of intra-abdominal space is routinely performed during every pediatric kidney transplantation. Particular attention is given to ensuring unobstructed venous outflow, which is verified multiple times intraoperatively using real-time ultrasound. If intraoperative findings suggest limited intra-abdominal capacity or venous congestion, surgical mobilization and adhesiolysis are carried out to increase available space. Should these measures prove insufficient, a temporary abdominal closure with an absorbable or biologic mesh and primary skin closure is performed according to institutional standard operating procedures. Using this approach, no cases of abdominal compartment syndrome occurred in the present cohort. The ureter was implanted using the Lich-Gregoir technique with a double-J catheter, which was usually removed 3–4 weeks after transplantation. Alternative techniques, including uretero-ureterostomy or uretero-cutaneostomy, were employed in patients with congenital urologic anomalies.

Standard immunosuppressive regimens included triple therapy with calcineurin inhibitors, mycophenolic acid, and corticosteroids. As a calcineurin inhibitor, cyclosporin (CsA) was routinely used until 2012; since then, tacrolimus (Tac) has been used. In patients with stable graft function, the target trough level ranges from 8 to 12 ng/mL (Tac) or 120–200 µg/L (CsA) during days 1–21 post-transplant, and 5–10 ng/mL (Tac) or 80–140 µg/L (CsA) from day 22 onward. Induction therapy was routinely administered only until around 2012 using anti-thymocyte globulin (ATG) or IL2-R antagonists (basiliximab or daclizumab) according to institutional protocol. Currently, induction therapy with basiliximab is reserved for cases of re-transplantation and/or the presence of preformed HLA antibodies. Immunosuppression was adjusted based on individual response and adverse events.

### Outcome measures

Primary endpoints included graft function, postoperative complications, and long-term graft and patient survival. Delayed graft function (DGF) was defined as the need for dialysis within the first post-transplant week. Initial non-function (INF) was defined as persistent dialysis dependence or a creatinine clearance ≤ 20 mL/min at three months. Primary non-function (PNF) was defined as permanent lack of graft function from the time of transplantation.

Episodes of rejection within the first year post-transplant were documented based on clinical suspicion and/or biopsy confirmation. Graft failure was defined as return to dialysis or preemptive re-transplantation. Kidney function was assessed using age-adjusted glomerular filtration rate (GFR).

### Statistical analysis

SPSS software, version 29.0 (SPSS Inc., Chicago, Illinois, USA) and GraphPad Prism software, version 10.4.0 (GraphPad Software, San Diego, California, USA) were used for statistical analysis and graphs.

Continuous variables were compared using Welch’s t-test. Categorical variables were analyzed using the chi-square test or Fisher’s exact test, as appropriate.

Graft and patient survival were estimated using Kaplan–Meier analysis and compared via the log-rank test. Graft survival was defined from the date of transplantation to either graft failure or patient death; a secondary analysis censored for death with a functioning graft. Patient survival was defined from the time of transplant to death, with censoring for loss to follow-up.

Statistical significance was set at a *p*-value < 0.05. Data are presented as mean ± standard deviation for normally distributed variables, median with interquartile range (IQR) for skewed variables, and as absolute numbers and percentages for categorical data.

## Results

### Study population

**Fig. 1:**
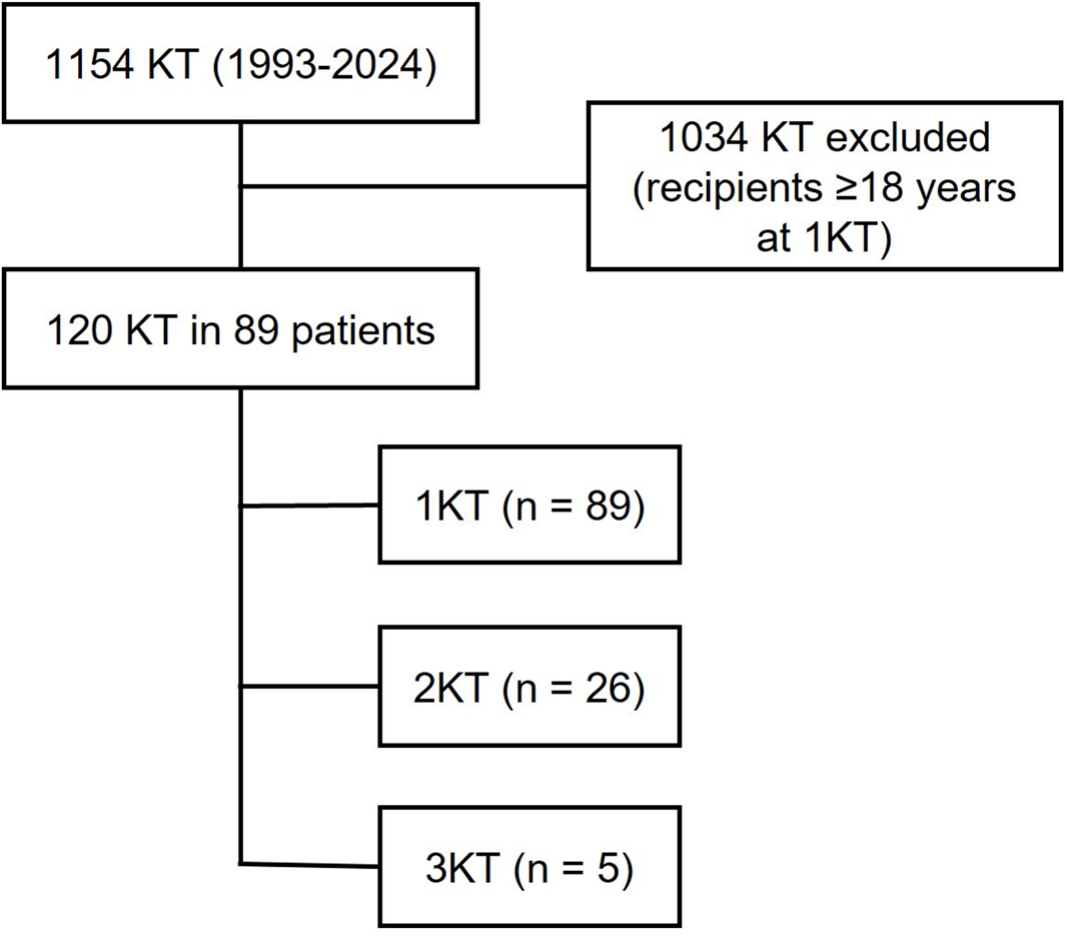
Flow chart of the study population. KT, kidney transplantation.

A total of 120 kidney transplantations were performed in 89 pediatric patients. Among these, 26 individuals (29.2%) received a second kidney transplant (2KT), and 5 (5.6%) underwent a third transplant (3KT) (Fig. 1). No en-bloc transplantations were performed. Overall median follow-up was 14.6 years (IQR 6.6, 23.5).

The median donor age at the time of first transplantation (1KT) was 33.0 years (IQR 15.0, 45.0), while the mean recipient age was 11.0 years (IQR 7.0, 14.5). Donor age showed a significant positive correlation with recipient age (*p* = 0.026).

Baseline characteristics of donors and recipients are summarized in Table 1. To ensure comparability, patients with graft loss after 1KT (1KT +) who subsequently underwent re-transplantation were analyzed in subgroup comparisons.

**Table 1:** Donor, recipient and transplant characteristics.

Variables	1KT (* n* = 89)	ReKT			*P*-value	*P*-value
		1KT + (*N* = 26)	2KT (*N* = 26)	3KT (*N* = 5)	ReKT	1–3KT
Donor						
Age, years (IQR)	33.0 (15.0, 45.0)	39.0 (18.3, 51.0)	47.0 (35.5, 54.3)	51.0 (30.5, 59.5)	0.518	0.013
Gender, female (%)	39 (43.8)	9 (34.6)	3 (11.5)	3 (60.0)	0.017	0.003
BMI, kg/m^2^	23.1 ± 4.5	23.7 ± 2.8	25.5 ± 3.9	24.6 ± 2.5	0.261	0.071
Donor type, LD (%)	15 (16.9)	4 (15.4)	5 (19.2)	0	0.563	0.570
Comorbidity (%)						
Hypertension	6 (6.7)	3 (11.5)	3 (11.5)	1 (20.0)	0.904	0.492
Diabetes mellitus	1 (1.1)	1 (3.8)	0	0	0.491	0.856
Cause of death CVA (%)	17 (19.1)	3 (11.5)	7 (26.9)	1 (20.0)	0.328	0.253
Recipient						
Age, years (IQR)	11.0 (7.0, 14.5)	11.0 (3.8, 13.3)	20.5 (12.75, 27.5)	21.0 (11.0, 32.0)	< 0.001	< 0.001
Gender, female, (%)	33 (37.1)	9 (34.6)		2 (40.0)	0.971	0.962
BMI, kg/m^2^	17.9 ± 3.9	17.5 ± 3.8	21.5 ± 4.8	21.6 ± 1.1	0.008	< 0.001
ESRD						
Congenital	24 (27.0)	3 (11.5)		0	0.932	0.533
FSGS	14 (15.7)	4 (15.4)		0		
Cystic	13 (14.6)	4 (15.4)		1 (20.0)		
Glomerulonephritis	4 (4.5)	2 (7.7)		1 (20.0)		
Interstitial nephritis	9 (10.1)	4 (15.4)		0		
Other	22 (24.7)	7 (26.9)		2 (40.0)		
Unknown	3 (3.4)	2 (7.7)		1 (20.0)		
Comorbidity (%)						
Hypertension	66 (74.2)	15 (57.7)	20 (76.9)	4 (80.0)	0.904	0.820
Time on dialysis, months (IQR)	16.8 (9.5, 31.1)	16.5 (8.6, 24.5)	48.0 (23.2, 86.0)	47.0 (22.8, 93.0)	< 0.001	< 0.001
Time on waiting list, months (IQR)	5.9 (2.4, 16.6)	4.6 (2.1, 9.0)	31.1 (5.4, 51.99	26.9 (1.2, 112.0)	0.005	0.011
Transplant						
Donor-recipient BSA ratio	1.80 ± 0.94	2.09 ± 1.03	1.34 ± 0.46	1.48 ± 0.65	0.011	0.069
CMV D + R- (%)	31 (34.8)	9 (34.6)	3 (11.5)	2 (40.0)	0.083	0.227
HLA mismatch ≥ 2 (%)	65 (73.0)	15 (57.7)	17 (65.4)	3 (60.0)	0.748	0.892
PRA positive (%)	11 (12.4)	5 (19.2)	17 (65.4)	4 (80.0)	< 0.001	< 0.001
CIT, hours	10.6 ± 5.4	9.6 ± 6.0	15.8 ± 9.3	15.5 ± 2.1	0.163	0.012
Total WIT, minutes	35.0 ± 16.6	27.5 ± 14.4	38.9 ± 32.2	46.0 ± 11.3	0.374	0.636
Surgery time, minutes	199.3 ± 56.8	218.3 ± 83.0	171.0 ± 56.3	387.3 ± 389.0	0.026	< 0.001
Intraoperative complications, all (%)	13 (14.6)	5 (19.2)	1 (3.8)	0	0.051	0.210
Induction therapy	13 (14.6)	5 (19.2)	14 (53.8)	2 (40.0)	0.021	< 0.001
ATG/ ILR2-RA (%)	0, 13 (100)	0, 5 (100)	1 (7.1), 13 (92.9)	1 (50.0), 1 (50.0)	0.309	0.102
Initial immunosuppression (%)						
CsA	54 (60.7)	22 (84.6)	10 (38.5)	3 (60.0)	0.007	0.191
Tac	33 (37.1)	2 (7.7)	15 (57.7)	2 (40.0)		
Tac + Ever	2 (2.2)	0	3 (11.5)	0		
Aza	2 (2.2)	2 (7.7)	0	0		
Total follow-up, years (IQR)	15.1 (6.9, 24.1)	24.9 (18.6, 28.2)	12.1 (3.4, 23.1)	17.4 (12.7, 23.6)	< 0.001	0.502

Aza, azathioprine; ATG, anti-thymocyte globulin; BMI, body mass index; BSA, body surface area; CIT, cold ischemia time; CMV D + /R−, cytomegalovirus status, donor + /recipient−; CsA, Ciclosporin A; CVA, cerebrovascular accident; ESRD, end-stage renal disease; Ever, everolimus; FSGS, focal segmental glomerulosclerosis; HLA, human leukocyte antigen; IL2-RA, Interleukin-2 receptor antagonist; LD, living donor; PD, peritoneal dialysis; PRA, panel reactive antibody; Tac, tacrolimus; WIT, warm ischemia time.

Living donor transplantations constituted 16.7% of all cases, with no significant differences across transplant groups (*p* = 0.570). The majority of living donors (90%) were biological parents.

Among recipients of a 2KT, 38.5% were under 18 years of age at the time of surgery, compared to 40% in the 3KT group. Duration of dialysis and time on the waiting list were significantly longer prior to 2KT and 3KT compared to 1KT + (dialysis: 16.5 vs. 48.0 vs. 47.0 months, *p* < 0.001; waiting list: 4.6 vs. 31.1 vs. 26.9 months, *p* = 0.005).

The proportion of panel reactive antibody (PRA)-positive patients increased significantly with successive transplants (19.2% vs. 65.4% vs. 80.0%, *p* < 0.001), while human leukocyte antigen (HLA) mismatch rates remained comparable.

Aorto-caval anastomoses were more frequently used in 1KT and became less common in repeat transplantations (aorta: 38.5% vs. 7.7% vs. 0%, *p* = 0.002; cava: 30.8% vs. 7.7% vs. 0%, *p* = 0.018). Especially, in children weighing < 20 kg (*n* = 21), grafts were usually implanted using aorto-caval anastomoses (vena cava: *n* = 18; aorta: *n* = 15).

In two re-transplantation cases, following transplant nephrectomy, the aorta and vena cava were reused for ipsilateral anastomosis (intraabdominal approach: *n* = 1; iliac fossa approach: *n* = 1). In all other cases, re-transplantation was performed via an extraperitoneal iliac fossa approach (ipsilateral 29.2%, contralateral 70.8%).

Surgical duration was significantly prolonged in 3KT (218 vs. 171 vs. 387 min, *p* = 0.026), whereas intraoperative complication rates did not differ significantly (19.2% vs. 3.8% vs. 0%, *p* = 0.051).

### Postoperative outcome

Early postoperative complications are summarized in Table 2. The most frequent early postoperative complication following 1KT was bleeding or hematoma (16.9%). Four vascular thromboses (two arterial, two venous) occurred post-transplant, whereby two patients underwent open surgical thrombectomy, and transplant nephrectomy was necessary in two cases.

**Table 2:** Post-operative outcome parameters, kidney function and survival.

Variables	1KT (N = 89)	ReKT			P-value	*P*-value
		1KT + (N = 26)	2KT (N = 26)	3KT (N = 5)	ReKT	1–3KT
Surgical outcome						
Time on ICU, days	14.4 ± 19.1	8.8 ± 8.0	12.1 ± 17.7	11.2 ± 7.46	0.745	0.843
Postoperative complications (%)						
Vascular occlusion or thrombosis	4 (4.5)	3 (11.5)	0	0	0.076	0.476
Bleeding/hematoma	15 (16.9)	2 (7.7)	5 (19.2)	2 (40.0)	0.333	0.504
Secondary wound healing	10 (11.2)	2 (7.7)	2 (7.7)	1 (20.0)	0.741	0.733
Lymphocele	6 (6.7)	2 (7.7)	2 (7.7)	0	0.733	0.805
Urine leakage	2 (2.2)	0	1 (3.8)	0	0.613	0.840
Clavien-Dindo ≥ 3b	25 (28.1)	10 (38.5)	9 (34.6)	3 (60.0)	0.419	0.369
Kidney function						
INF (%)	3 (3.4)	1 (3.8)	3 (11.5)	2 (40.0)	0.124	0.005
DGF (%)	16 (18.0)	4 (15.4)	4 (15.4)	1 (20.0)	0.901	0.943
Rejection treated, 1st year (%)	33 (37.1)	8 (30.8)	8 (30.8)	2 (40.0)	0.977	0.882
Biopsy performed	29 (87.9)	6 (75.0)	7 (87.5)	2 (100)	0.731	0.876
Histologically confirmed	22 (66.7)	5 (62.5)	5 (62.5)	2 (100)	0.802	0.258
Treatment						
High-dose corticosteroids	21 (63.6)	6 (75.0)	4 (50.0)	1 (50.0)	0.802	0.431
Adjustment of IS	16 (48.5)	5 (62.5)	3 (37.5)	1 (50.0)		
ATG	0	0	1 (12.5)	0		
Plasmapheresis	0	0	1 (12.5)	0		
GFR (mL/ min)						
POD 7	53.5 ± 48.3	51.9 ± 39.1	43.0 ± 42.0	23.8 ± 19.9	0.452	0.357
POD 14	62.5 ± 49.6	53.4 ± 34.1	60.6 ± 30.6	38.5 ± 21.3	0.526	0.680
POM 1	72.9 ± 40.1	74.0 ± 32.0	60.4 ± 32.9	46.1 ± 17.0	0.255	0.224
POM 3	60.9 ± 26.9	58.7 ± 26.7	56.7 ± 23.0	57.6 ± 9.7	0.973	0.825
POM 6	61.3 ± 28.4	60.4 ± 23.5	59.0 ± 19.8	56.2 ± 7.3	0.948	0.916
Hospitalisation, days	37.4 ± 20.7	37.3 ± 19.8	31.7 ± 18.4	54.8 ± 43.5	0.149	0.123
Long-term-outcome						
Death-censored graft survival (%)						
1-year	78 (88.7)	17 (65.4)	20 (83.8)	5 (100.0)	0.124	0.592
5-year	58 (79.6)	13 (50.0)	13 (79.2)	4 (80.0)	0.086	0.965
10-year	39 (63.7)	8 (30.8)	7 (56.7)	1 (20.0)	0.105	0.190
Death-censored graft survival, years	14.8 ± 1.5	6.9 ± 1.6	13.5 ± 2.6	7.4 ± 1.6	0.065	0.134

ATG, anti-thymocyte globulin; DGF, delayed graft function; ICU, intensive care unit; INF, initial non-function; IS, immunosuppression; POD, postoperative day, POM, postoperative month.

There were no significant differences in postoperative complication rates—including major complications (Clavien-Dindo ≥ grade 3b)—between transplant groups (*p* = 0.419).

Overall, delayed graft function (DGF) occurred in 17.5% of cases, and acute rejection within the first year was observed in 35.8%.

Rates of DGF (*p* = 0.901) and initial non-function (INF, *p* = 0.124) were also comparable. No case of PNF was observed.

Kidney function within six months post-transplant, as measured by glomerular filtration rate (GFR), did not differ significantly among the groups.

Hospital length of stay was longest after 3KT (37.3 vs. 31.7 vs. 54.8 days), though the difference was not statistically significant (*p* = 0.149).

### Long-term outcome and survival

During follow-up, 21 patients (17.5%) received treatment for cytomegalovirus (CMV) infection or viremia, and 14 patients (11.7%) for polyomavirus (PV)-virus infection or viremia. There was no significant difference between the groups (*p* = 0.656, and *p* = 0.631, respectively). CMV treatment included antiviral therapy (76.2%) and adjustment of immunosuppression (23.8%). PV treatment included reduction (50.0%) or change (50.0%) of immunosuppression. Among 14 patients with PV viremia, 11 developed rejections, with graft loss in 3 cases.

**Fig. 2:**
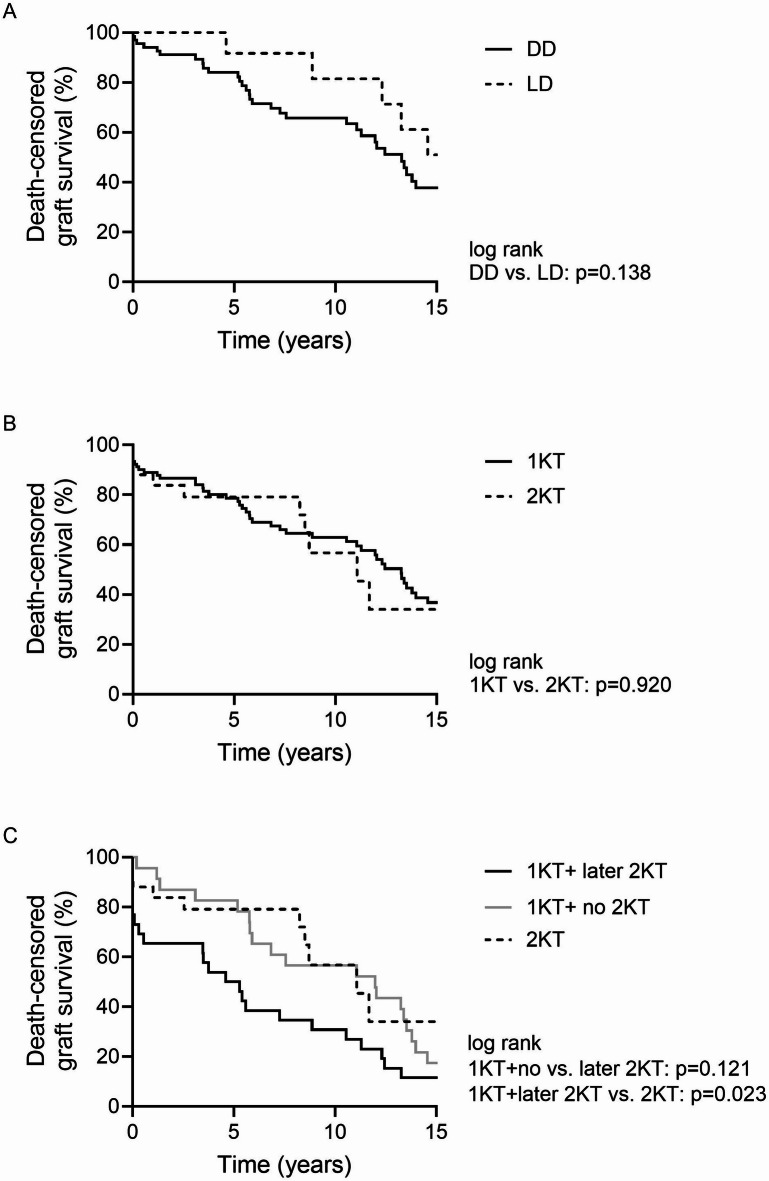
Death-censored kidney graft survival in (**A**) primary kidney transplantation (1KT) after deceased and living donation, (**B**) primary (1KT) and second kidney transplantation (2KT), and (**C**) primary kidney transplantation and later graft failure (1KT +) and second kidney transplantation (2KT).

Kaplan–Meier survival curves are shown in Fig. 2. Survival outcomes did not differ significantly between deceased donor (DD) and living donor (LD) transplants (*p* = 0.138) (Fig. 2A).

The 1-, 5-, and 10-year graft survival following 1KT were comparable to those after 2KT (1KT vs. 2KT: 88.7%, 79.6%, and 63.7% vs. 83.8%, 79.2%, and 56.7%, respectively; *p* = 0.920) (Fig. 2B).

In the re-transplantation group, mean graft survival was 6.9 ± 1.6 years for 1KT + , 13.5 ± 2.6 years for 2KT, and 7.4 ± 1.6 years for 3KT (*p* = 0.065) (Fig. 2C).

Kaplan–Meier analysis demonstrated significantly improved graft survival after 2KT compared to 1KT + (*p* = 0.023).

Long-term patient survival was excellent, with 10-, 20-, and 30-year survival rates of 100, 90.8, and 90.8%, respectively. Median patient survival was 44.3 ± 1.2 years, with no significant differences across transplant groups.

Five patients died during the follow-up period. Reported causes of death included COVID-19, pneumonia, sepsis, Burkitt’s lymphoma, and one case of unknown etiology. None of these patients were listed for re-transplantation.

### Causes of graft loss and re-transplantation

Causes of graft loss are detailed in Table 3. The most common causes of graft loss following 1KT were chronic allograft nephropathy (34.7%) and acute or chronic rejection (32.7%). Non-adherence, primarily among young adults, accounted for 6.1% of graft losses.

**Table 3:** Causes of kidney graft loss and patient characteristics.

Variables	1KT (N = 89)	ReKT			*P*-value	*P*-value
		1KT + (N = 26)	2KT (N = 26)	3KT (N = 5)	ReKT	1–3KT
Cause of graft loss (%)						
Death	1 (1.1)	0	1 (3.9)	0		
Graft failure, censored	49 (55.1)	26 (100)	10 (38.5)	5 (100)		
Cause of graft failure (%)						
Vascular	5 (10.2)	5 (19.2)	0	0	0.193	0.436
Acute or chronic rejection	16 (32.7)	10 (34.6)	3 (30.0)	1 (20.0)	0.692	0.641
Recurrence of kidney disease	3 (6.1)	2 (7.7)	1 (10.0)	0	0.776	0.750
Chronic allograft nephropathy	17 (34.7)	7 (26.9)	3 (30.0)	1 (20.0)	0.918	0.784
Infection	1 (2.0)	0	1 (10.0)	3 (60.0)	< 0.001	< 0.001
Other	3 (6.1)	2 (7.7)	0	0	0.545	0.612
Unknown	5 (10.2)	1 (3.8)	0	0	0.744	0.436
Re-listing (%)	32 (65.3)		8 (80.0)	2 (40.0)		
Reason no re-listing						
Medical contraindication	5 (10.2)		1 (10.0)	0		
Insufficient adherence	3 (6.1)		0	0		
Patient decision	1 (2.0)		0	0		
Re-listing planned	2 (4.1)		0	2 (40.0)		
Death	1 (2.0)		1 (10.0)	0		
Unknown	5 (10.2)		0	1 (20.0)		

Graft loss due to recurrence of the underlying kidney disease was observed in 6.3% of cases. Patients receiving re-transplantation were significantly younger at the time of graft loss (16.9 ± 10.6 vs. 23.3 ± 8.0 years, *p* = 0.026), and more frequently under 18 years of age (57.7% vs. 22.7%, *p* = 0.014).

Of the 49 patients with graft failure after 1KT, 11 (22.4%) were not re-listed for transplantation. The most common reasons included medical contraindications (e.g., chronic infections, malignancy, cardiopulmonary disease), lack of adherence, or patient preference.

All patients with vascular-related graft loss subsequently received re-transplantation. One patient was excluded after 2KT due to high sensitization. In the 3KT group, infections were the leading cause of graft loss (60%, *p* < 0.001).

## Discussion

This retrospective study confirms that repeated kidney transplantation (ReKT) in pediatric patients is both feasible and effective, yielding outcomes that are comparable to—or in some cases better than—those of primary transplantation.

In our cohort, approximately 80% of patients had functioning grafts five years after their first kidney transplant (1KT), consistent with international benchmarks^2,10,11^. Chronic allograft nephropathy and immunological rejection remained the predominant causes of graft loss, aligning with previous reports^8^.

Notably, nearly half of the patients experiencing graft failure underwent a second transplant, typically within five years. This reflects favorable access to re-transplantation within our cohort. A substantial proportion of ReKT recipients were under 18 years of age at the time of re-listing (57.7%), suggesting that early graft failure, fewer comorbidities, and timely referral may facilitate access to and success of re-transplantation.

Patients experiencing vascular complications after 1KT were more likely to receive ReKT. Donor-recipient body size mismatch has been implicated in vascular thrombosis and primary graft non-function in pediatric transplantation^12^. To mitigate these risks, our center employed aorto-caval anastomoses in smaller children—a technique that became less necessary as patient size increased with age.

Interestingly, patients in the 2KT group demonstrated significantly better graft survival compared to the 1KT + group, despite a higher prevalence of sensitization (PRA positivity) and longer dialysis durations. This finding is consistent with a multicenter study by van Arendonk et al., which also reported favorable outcomes following 2KT^11^. Several factors may contribute to this improvement, including selective re-transplantation based on patient survival, adherence, and immunologic profile. Technological advances in HLA typing and crossmatching have also enabled more precise donor-recipient pairing, which likely contributes to improved graft longevity. Younger age at the time of graft loss has additionally been associated with increased access to re-transplantation and better post-ReKT outcomes^11^.

In our cohort, some patients with graft failure did not undergo re-transplantation due to medical contraindications, non-adherence, or personal choice. At our center, every case of graft failure is routinely presented in an interdisciplinary and interprofessional transplant conference to analyze contributing factors and plan further management. Re-evaluation for potential re-listing is a center-wide standard unless patients decline participation. This re-evaluation comprises a comprehensive medical assessment and adherence screening by a mental health professional. Re-listing and re-transplantation were offered only to patients who fulfilled eligibility criteria and had no major concerns regarding expected adherence, in line with the principles laid out in the German Transplantation Act (Transplantationsgesetz, TPG) and the guidelines of the German Medical Association (Bundesärztekammer, BÄK) regarding urgency and likelihood of success. Because both comorbidity and adherence are dynamic, eligibility was re-assessed during the waiting period to ensure that re-transplantation remained medically and ethically justified.

Living donor (LD) transplantations accounted for 16.7% of procedures in our cohort—slightly below European averages. A living donor program was established at our center in 2005. Following its introduction, the rate of LD KT increased over time (1995–2004: 10.2%, 2005–2014: 22.6%, 2015–2024: 23.6%; *p* = 0.201). Although not statistically significant, LD recipients in our study demonstrated a trend toward improved graft survival. Previous studies have shown that LD is associated with superior outcomes due to better HLA compatibility, shorter cold ischemia time, and healthier donor profiles^10,13^. Importantly, this survival benefit appears to persist across both primary and repeat transplantation settings, irrespective of prior donor type. Notably, cumulative graft survival was similar regardless of whether the donor sequence was deceased-to-living or living-to-deceased^14^.

Nonetheless, the decision to pursue a living or deceased donor graft must be individualized. For instance, it has been suggested that highly sensitized pediatric patients (PRA > 80%) may benefit from prioritization for deceased donor grafts, given their accelerated access to organ offers through allocation systems^15^. Our sample size was insufficient to support detailed subgroup analysis of donor sequence and its effect on ReKT outcomes.

Collectively, these findings affirm the feasibility of ReKT in pediatric ESRD patients and underscore the importance of timely referral, optimal donor selection, and individualized surgical planning.

### Limitations

This study has several limitations. First, the retrospective and non-randomized design inherently limits causal inference. Second, the small sample size within subgroups—particularly for third transplants—reduces the statistical power to detect significant differences. Third, the extended study period posed challenges for consistent data collection and uniform documentation practices over time.

### Conclusion

Second kidney transplantation in pediatric patients is feasible and associated with favorable long-term outcomes—occasionally surpassing those of initial transplantation. Although technically more complex, third kidney transplantation remains a viable option with acceptable risk in selected patients. Future prospective multicenter studies are warranted to validate these findings and refine clinical strategies for pediatric re-transplantation.

## Data Availability

The datasets generated during and/or analyzed during the current study are available from the corresponding author on reasonable request.

## Abbreviations

1KT: First kidney transplantation
2KT: Second kidney transplantation
3KT: Third kidney transplantation
ATG: Anti-thymocyte globulin
BSA: Body surface area
BMI: Body mass index
CIT: Cold ischemia time
CMV: Cytomegalovirus
CVA: Cerebrovascular accident
DD: Deceased donor
DGF: Delayed graft function
ESRD: End-stage renal disease
FSGS: Focal segmental glomerulosclerosis
GFR: Glomerular filtration rate
GN: Glomerulonephritis
HLA: Human leukocyte antigen
ICU: Intensive care unit
INF: Initial non-function
KT: Kidney transplantation
LD: Living donor
PNF: Primary non-function
PV: Polyomavirus
POD: Postoperative day
POM: Postoperative month
PRA: Panel reactive antibody
ReKT: Repeated kidney transplantation
WIT: Warm ischemia time
